# Supplementary material for: Plasticity of the Mycobacterium tuberculosis respiratory chain and its impact on tuberculosis drug development
Source: Nat Commun. 2019 Oct 31;10:4970. doi: 10.1038/s41467-019-12956-2 (PMC6823465; doi:10.1038/s41467-019-12956-2)
Supplement: Supplementary file 1 — Supplementary Information [file 41467_2019_12956_MOESM1_ESM.pdf]

Supplementary Information

Plasticity of the *Mycobacterium tuberculosis* respiratory chain and its impact on tuberculosis drug development

Tiago Beites, Kathryn O'Brien, Divya Tiwari, Curtis A. Engelhart, Shaun Walters, Jenna Andrews, Hee-Jeong Yang, Michelle L. Sutphen, Danielle M. Weiner, Emmanuel K. Dayao, Matthew Zimmerman, Brendan Prideaux, Prashant V. Desai, Thierry Masquelin, Laura E. Via, Véronique Dartois, Helena I. Boshoff, Clifton E. Barry III, Sabine Ehrt, Dirk Schnappinger

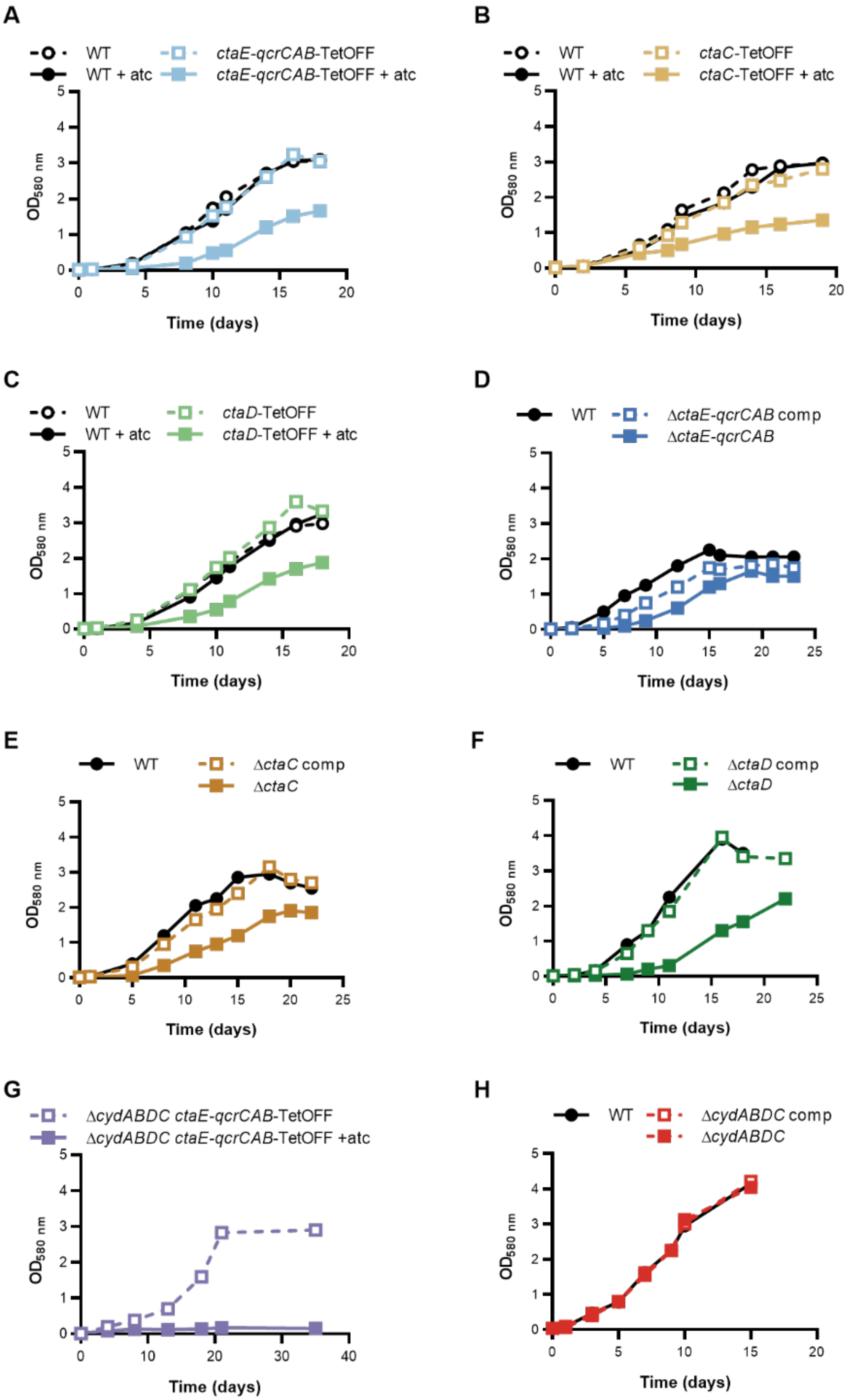

Supplementary Fig. 1. Cytochrome *bd* oxidase and cytochrome *bc*$_1$-*aa*$_3$ oxidase are individually dispensable and synthetically lethal. Growth curves of conditional knockdown mutants *ctaE-qcrCAB*-TetOFF (A), *ctaC*-TetOFF (B), *ctaD*-TetOFF (C), Δ*cydABDC ctaE-qcrCAB*-TetOFF (G), and deletion mutants Δ*ctaE-qcrCAB* (D), Δ*ctaC* (E), Δ*ctaD* (F), Δ*cydABDC* (H). All strains were grown in regular 7H9. Data are representative of at least two independent experiments. "Comp" stands for complemented. Source data are provided as a Source Data file.

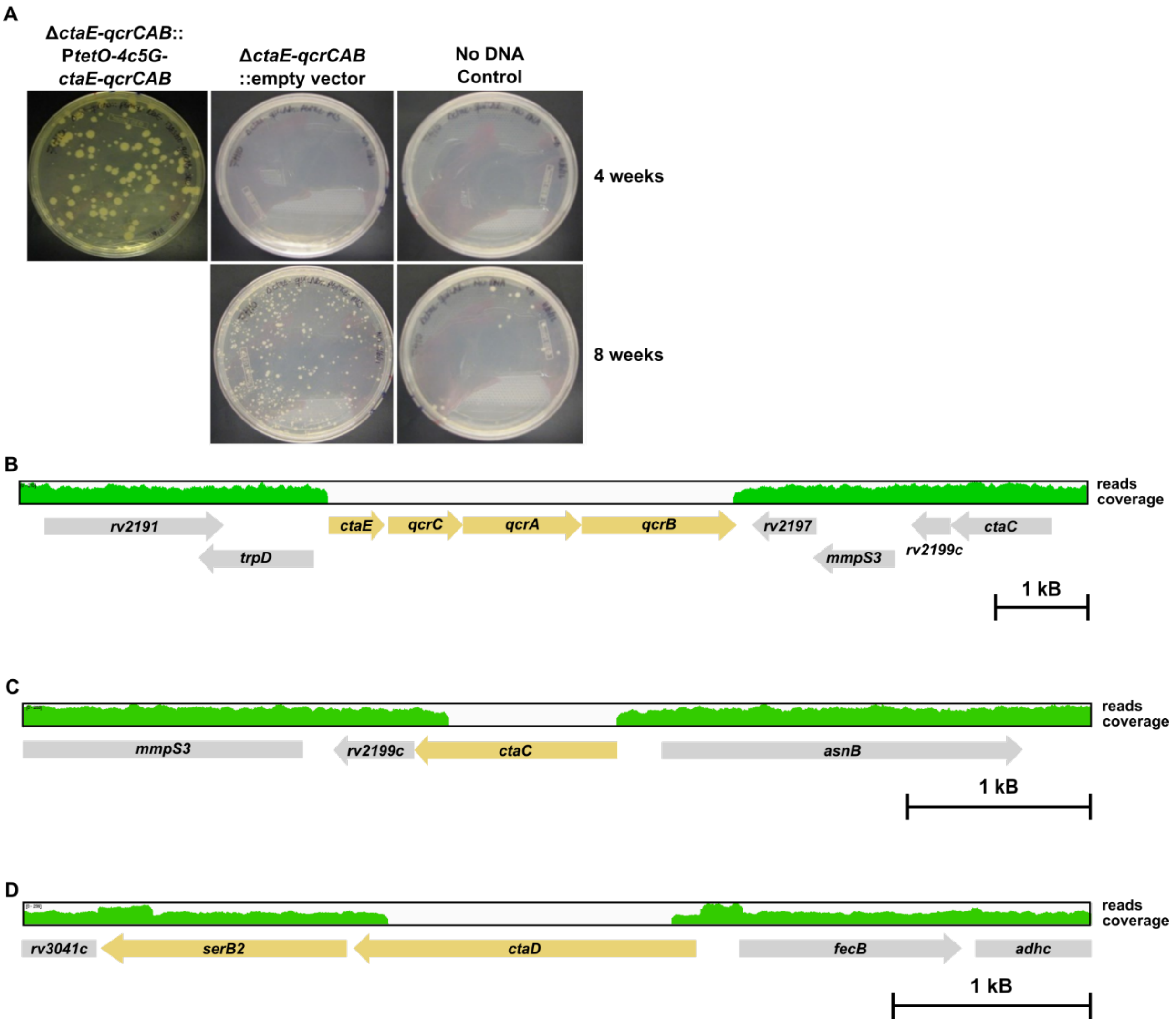

Supplementary Fig. 2. Construction of mutants in which subunits of the cytochrome *bc1-aa3* complex have been deleted. **(A)** Isolation of *Mtb* Δ*ctaE-qcrCAB*. We first constructed a merodiploid containing a second copy of *ctaE-qcrCAB* integrated into the attachment site of the phage L5 (attL5). Next, the WT copy was deleted resulting in *Mtb* Δ*ctaE-qcrCAB*::P*ctaE-qcrCAB*. To eliminate the remaining copy of *ctaE-qcrCAB*, the *ctaE-qcrCAB* expression plasmid in the attL5 was swapped against an "empty" vector. Swaps with another expression plasmid (containing *ctaE-qcrCAB* under the control of the strong promoter Ptet0-4C5G) were performed as a control. Transformants were spread on 7H10 agar plates and photographed after 4 and 8 weeks of incubation at 37 °C. (**B, C, D**) Whole genome sequencing coverage showing gene deletion of the wild-type loci of *ctaE-qcrCAB*, *ctaC*, and *ctaD*. The reads corresponding to *serB2* originate from a copy inserted at the att-L5 site.

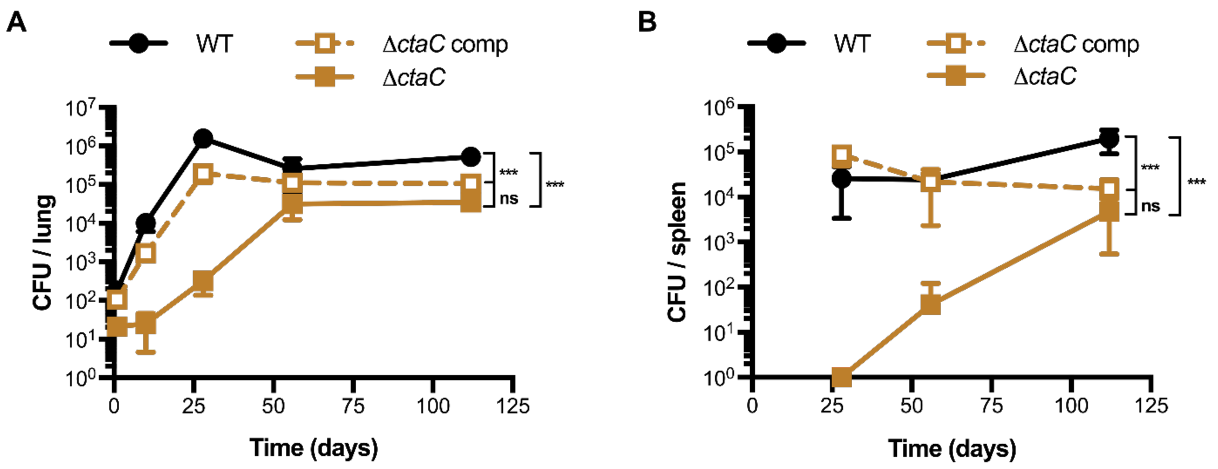

Supplementary Fig. 3. The cytochrome *bc*$_1$-*aa*$_3$ complex is dispensable for growth and persistence of *Mtb* in mice. Growth and persistence of Δ*ctaC* in mouse lungs **(A)** and spleens **(B)**. Data are averages of CFUs from at least three mice per time point. Error bars correspond to standard deviation. "Comp" stands for complemented. Statistical significance was assessed by one-way ANOVA followed by post hoc test (Tukey test; GraphPad Prism). *** P<0.001; ns - not significant. Source data are provided as a Source Data file.

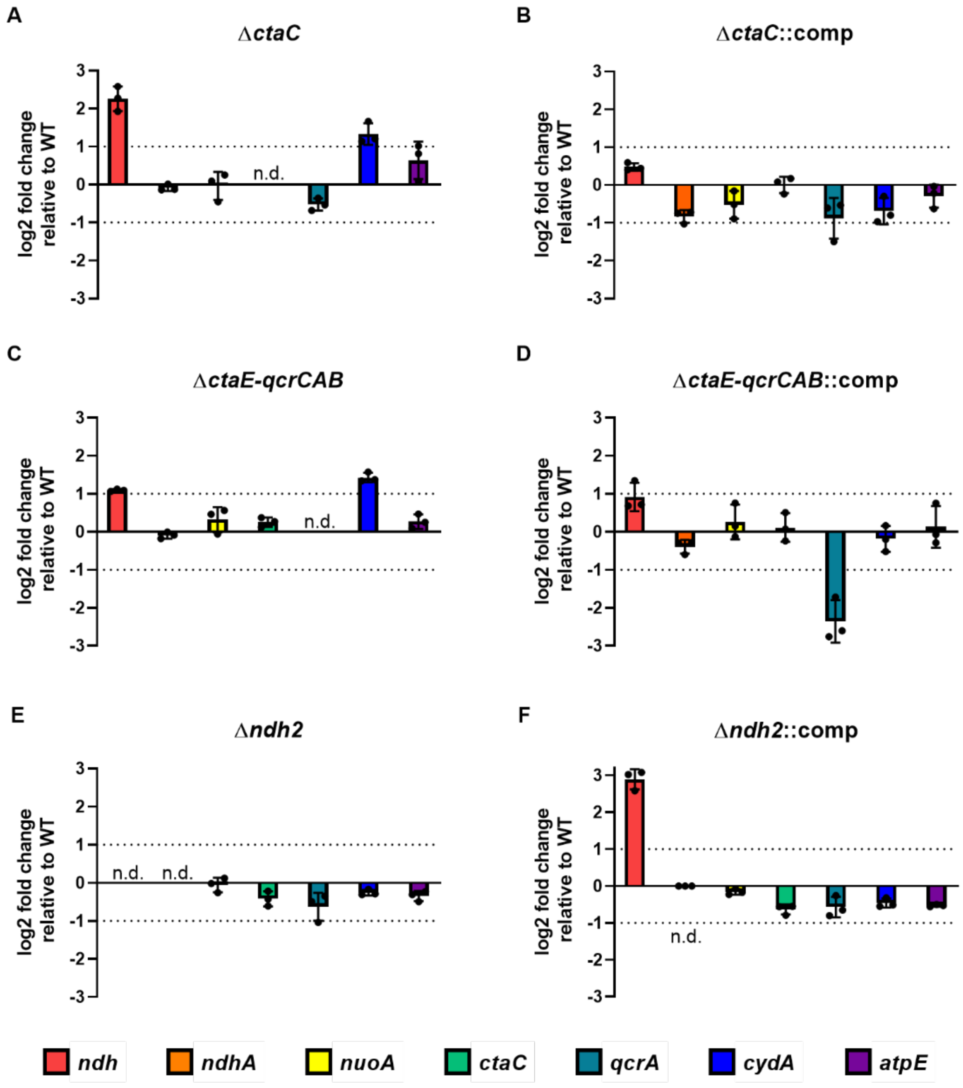

Supplementary Fig. 4. Transcription profiles of genes encoding RC enzymes. Δ*ctaC* **(A)**, Δ*ctaC*::comp **(B)**, Δ*ctaE-qcrCAB* **(C)**, Δ*ctaE-qcrCAB*::comp **(D)** and parental strain were grown in 7H9 until mid-exponential phase. Δ*ndh-2* **(E)**, Δ*ndh-2*::comp **(F)**, and parental strain were grown in modified Sauton's minimal medium until mid-exponential phase. *sigA* was used as reference gene. qPCR data is represented as log_2 fold change relative to WT, and correspond to data from 3 independent experiments. Error bars correspond to standard deviation. "Comp" stands for complemented. Source data are provided as a Source Data file.

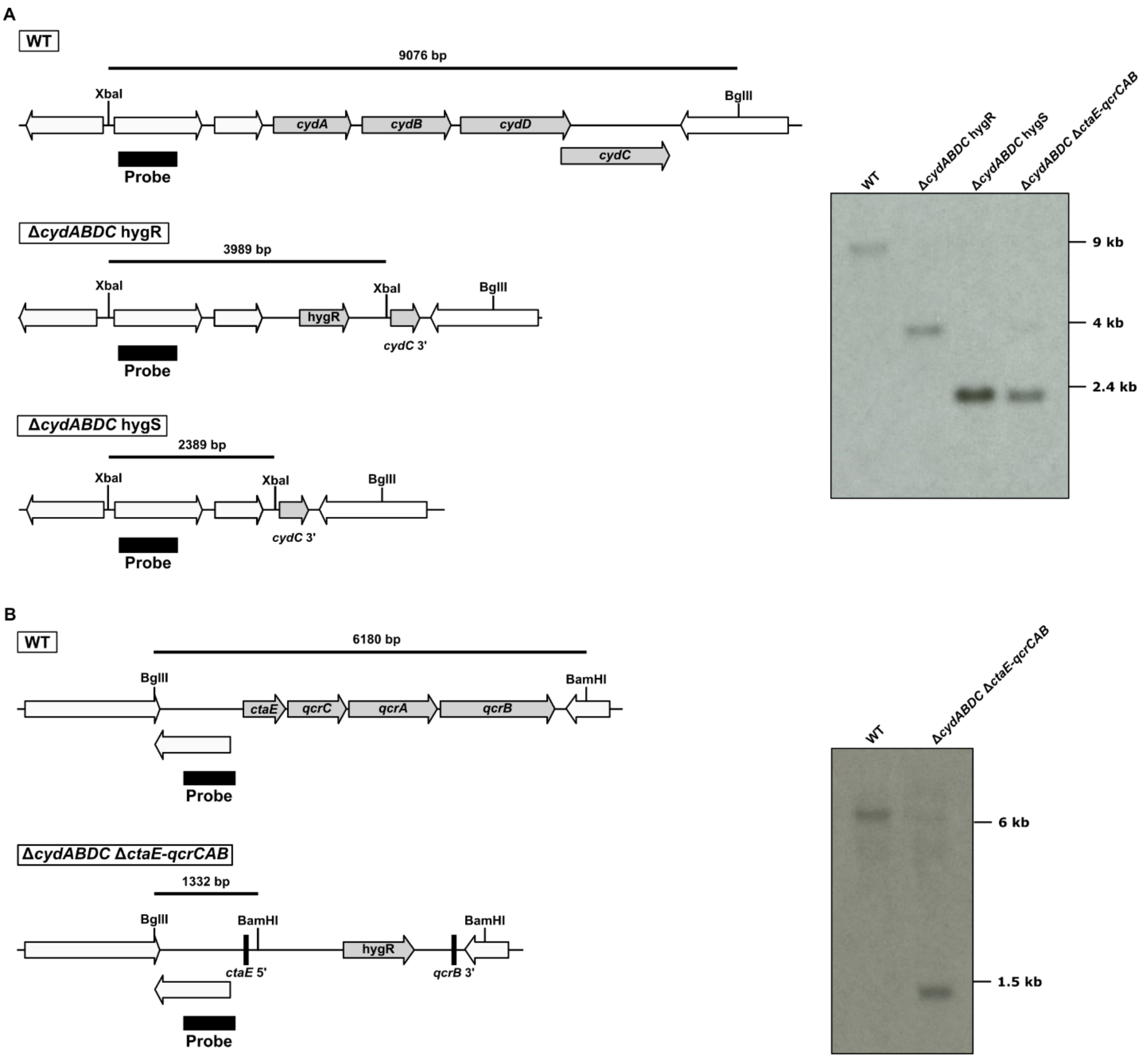

Supplementary Fig. 5. Southern blots verifying Δ*cydABDC* Δ*ctaE-qcrCAB*::*ctaE-qcrCAB*-TetOFF. (A, B) Southern blots for sequential gene deletion steps. First the *cydABDC* operon was substituted by a hygromycin resistance cassette (hygR). Next, hygR was eliminated rendering Δ*cydABDC* hygromycin sensitive (hygS). This strain was then transformed with a plasmid containing a second copy of *ctaE-qcrCAB* under the control of a Tet-OFF system that integrated into attL5. Finally, *ctaE-qcrCAB* wild-type locus was replaced by hygR. Source data are provided as a Source Data file.

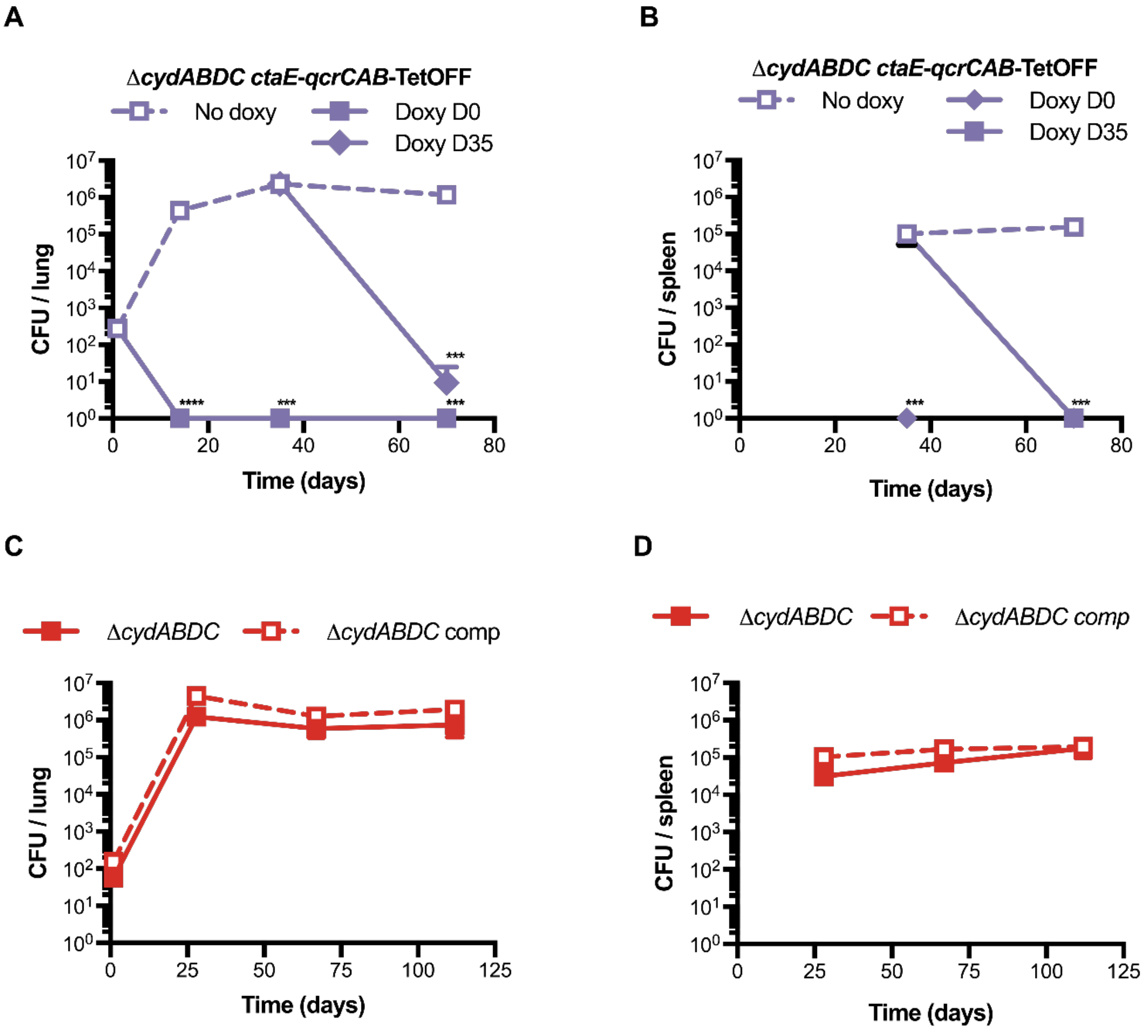

Supplementary Fig. 6. Functional redundancy with cytochrome *bd* oxidase causes dispensability of the cytochrome bc_1-aa_3 complex for growth and persistence of *Mtb* in mice. Growth and persistence of Δ*cydABDC ctaE-qcrCAB*-TetOFF **(A, B)** and Δ*cydABDC* **(C, D)** in mouse lungs and spleens. Data are averages of CFUs from at least four mice per time point. Error bars correspond to standard deviation. "Comp" stands for complemented. Statistical significant differences between control and treated situations were assessed by unpaired, two-tailed *t*-test. No statistical differences were observed in **(C)** and **(D)**. *** P<0.001; **** P<0.0001. Source data are provided as a Source Data file.

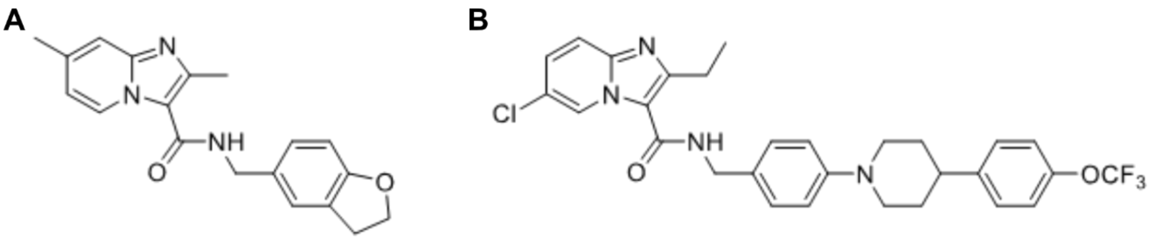

Supplementary Fig. 7. Conserved pharmacophore in ND-10885 and Q203. Structures of ND-10885 **(A)** and Q203 **(B)** the conserved imidazo[1,2-*a*]pyridine-3-carboxamide core.

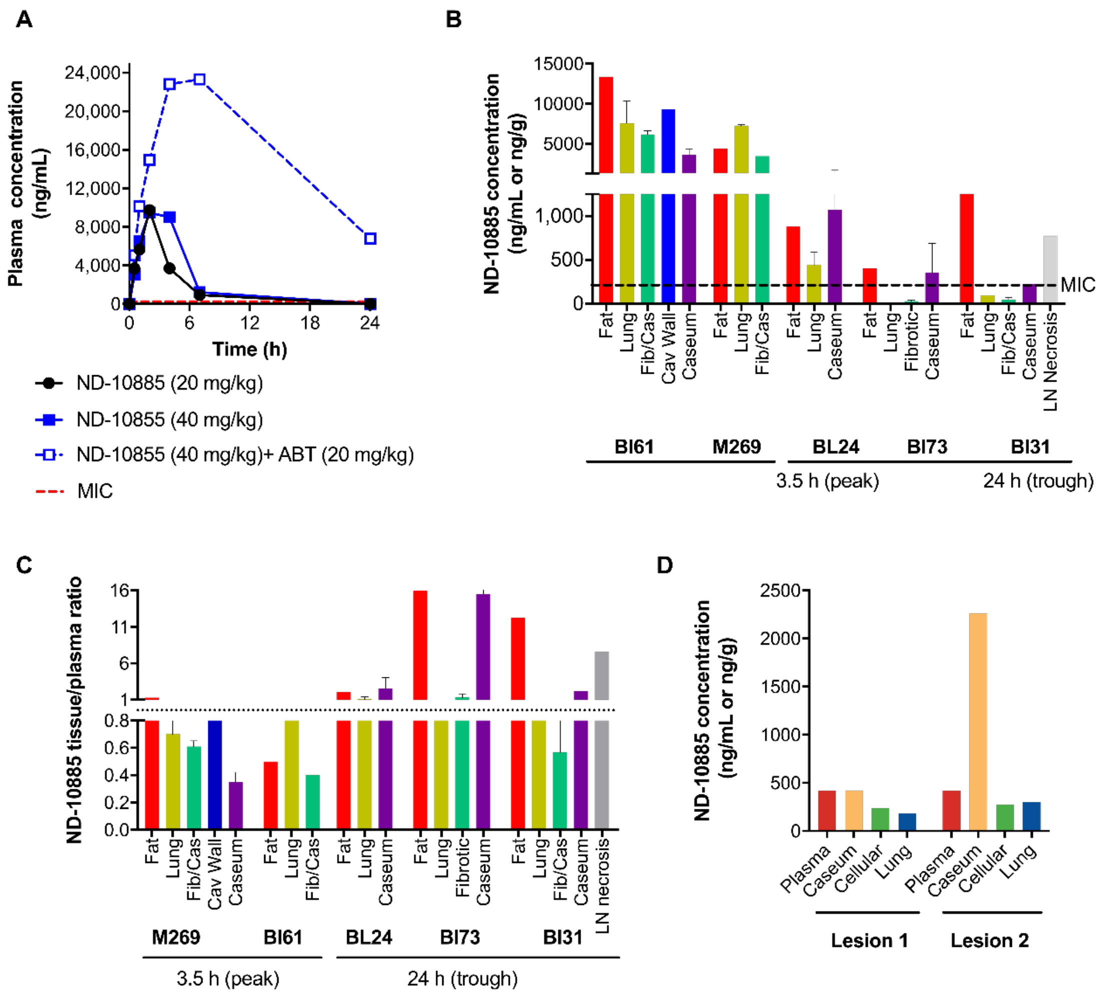

Supplementary Fig. 8. Plasma pharmacokinetics and tissue distribution of ND-10885 in marmosets. (A) Plasma pharmacokinetics of ND-10885 following a single oral dose in uninfected marmosets, with and without co-ABT (1-aminobenzotriazole) administered orally as described. Absolute quantitation (B) and relative distribution (C) of ND-10885 in adipose and lung tissue, and in distinct lesion compartments, in TB infected marmosets receiving 12.5 mg/kg ND-10885 twice daily (at treatment completion). BI31, BL24 and BI73 did not receive the second daily dose of ND-10885 on the day of necropsy, thus the 24h levels are lower than on previous days when they received two daily doses. (D) Spatial quantitation of ND-10885 in lung tissue, cellular layers, and caseous foci of two closed granulomas, relative to plasma. ND-10885 concentrations were measured in laser-captured microdissected areas from thin tissue sections. Both lesions and surrounding lung tissue were excised from marmoset BL24, which received the last dose of 12.5 mg/kg ND-10885 24h prior to necropsy. Error bars correspond to standard deviation. Fib/Cas: closed caseous fibrotic granuloma; Cav: cavity; LN: lymph node. Source data are provided as a Source Data file.

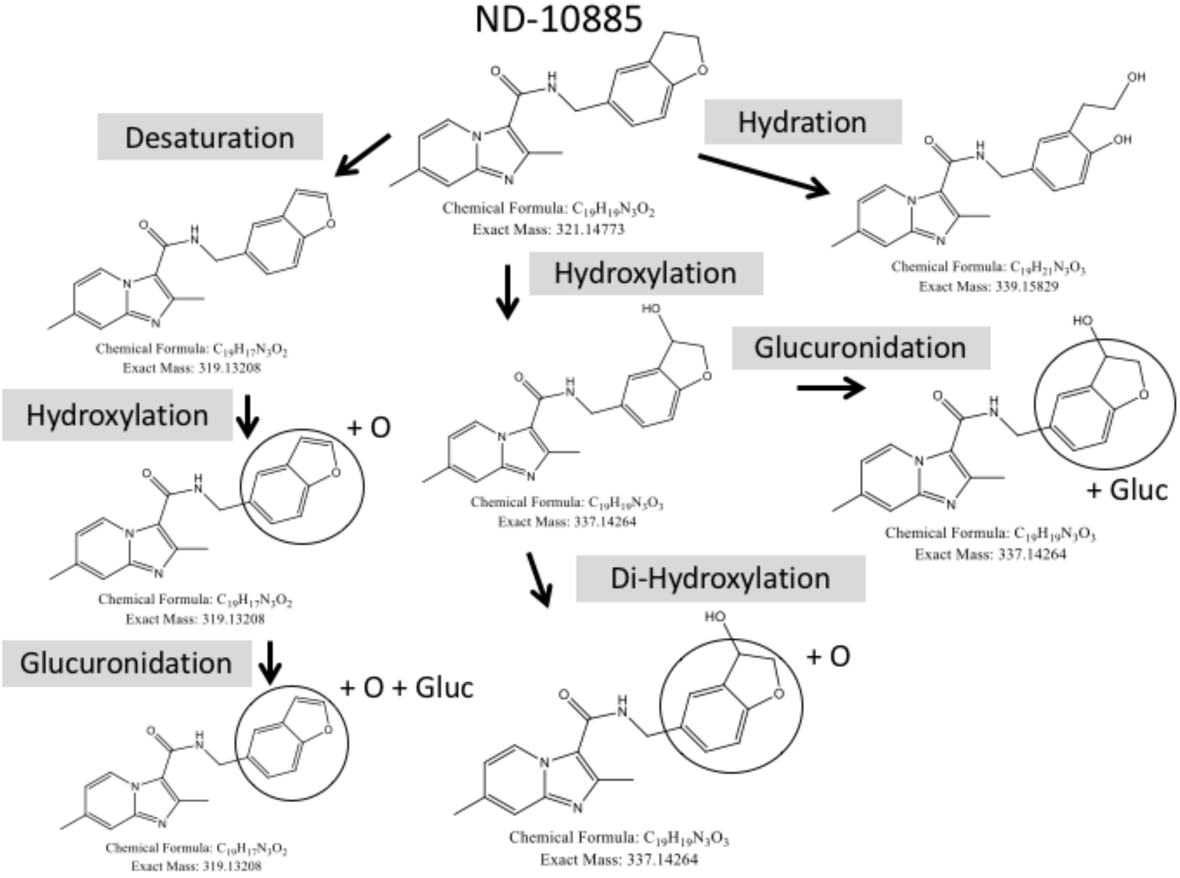

Supplementary Fig. 9. Biotransformation of ND-10885. Metabolites of ND-10885 detected and identified in marmoset plasma following a single oral dose. Metabolites were extracted from plasma using protein precipitation. Mass spectrometry analysis was performed on a Thermo Q-Exactive® HRMS mass spectrometer using Xcalibur 3.0® data acquisition software. HPLC separation was performed on an Ultimate 3000® HPLC system with an Agilent SB-C8® 2.1 × 30 mm, 3.5-[illegible]m column using a reverse phase gradient. Compound Discoverer 2.0® software was used to data mine the high resolution full mass spectrum for metabolites and identify locations for metabolism using a mass accuracy of 10 ppm. Gluc.: glucuronidation.

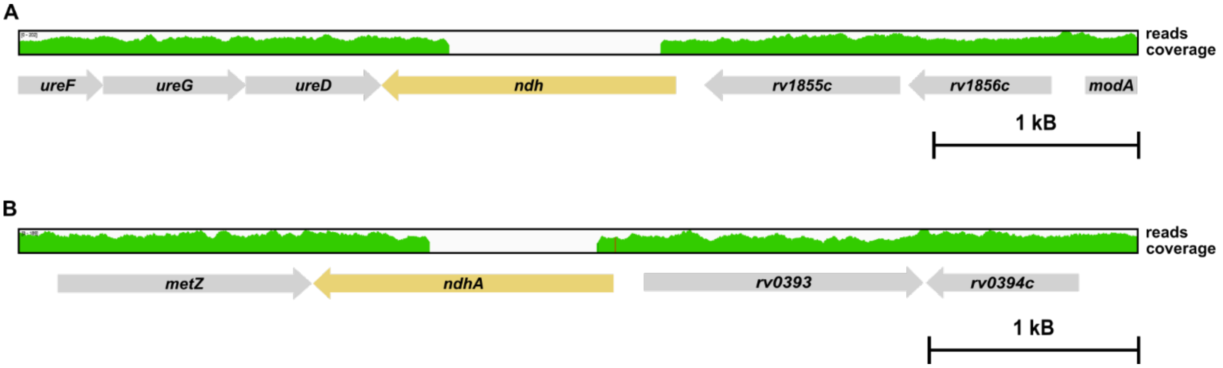

Supplementary Fig. 10. Verification of Δ*ndh*-2. Whole genome sequencing coverage showing gene deletion of the wild-type loci of *ndh* **(A)** and *ndhA* **(B)**.

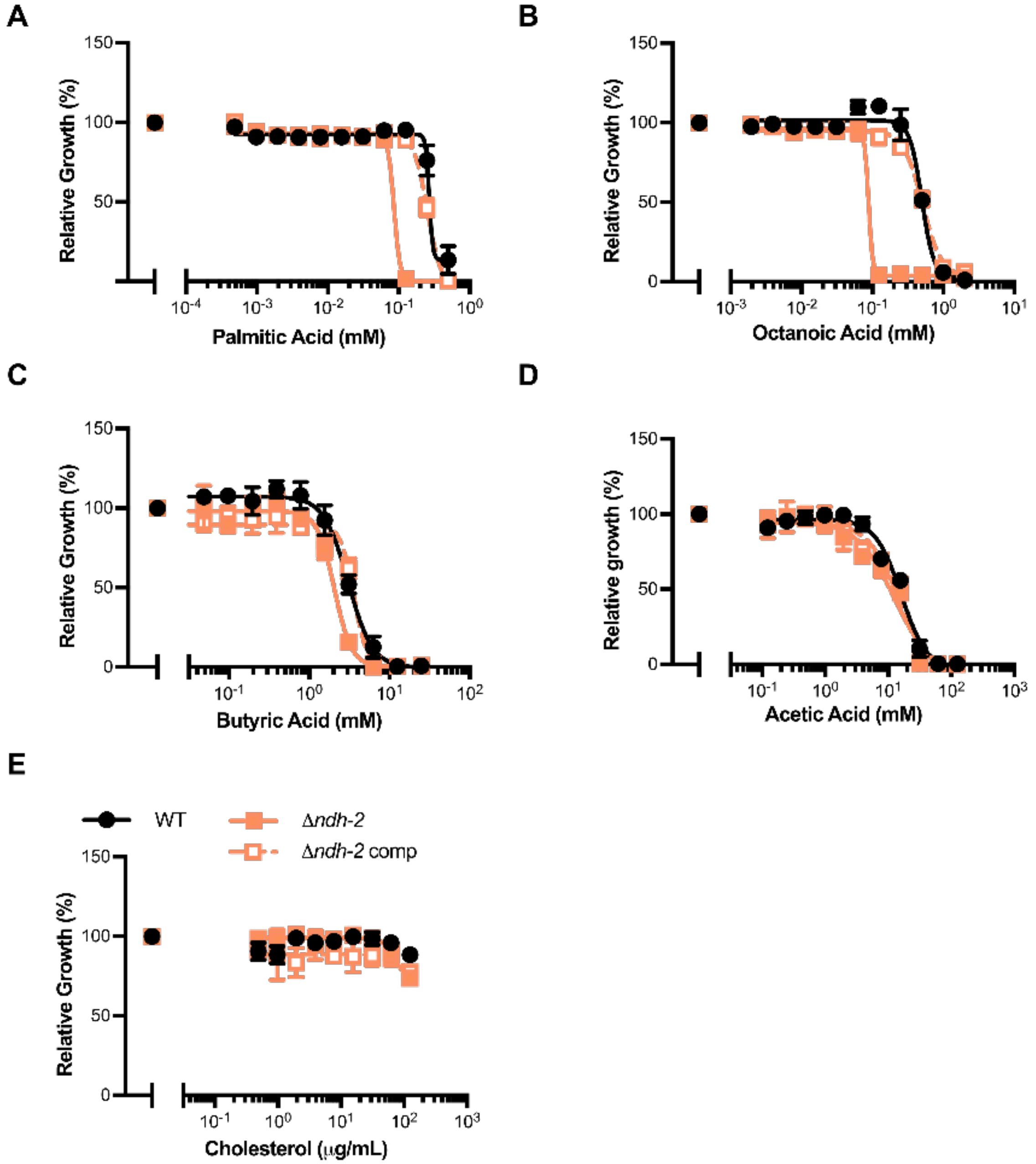

Supplementary Fig.11. Growth of Δ*ndh-2* with different fatty acids and cholesterol. Wild-type, Δ*ndh-2*, and complemented Δ*ndh-2* were grown in fatty acid free Sauton's minimal medium supplemented with increasing concentrations of palmitic acid **(A)**, octanoic acid **(B)**, butyric acid **(C)**, acetic acid **(D)** and cholesterol **(E)**. Data are average of triplicates and represent at least two independent experiments. Error bars correspond to standard deviation. "Comp" stands for complemented. Source data are provided as a Source Data file.

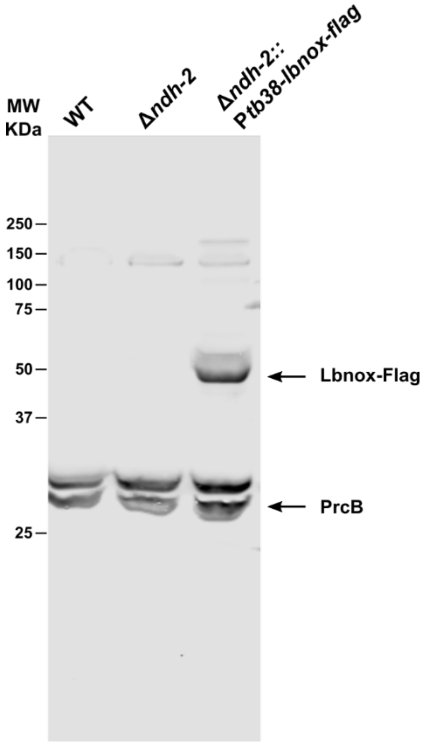

Supplementary Fig. 12. Detection of LbNox-Flag expression in Δ*ndh-2*. LbNox-Flag was detected with an anti-flag antibody. PrcB was used as a loading control. MW – molecular weight. Source data are provided as a Source Data file.

A B

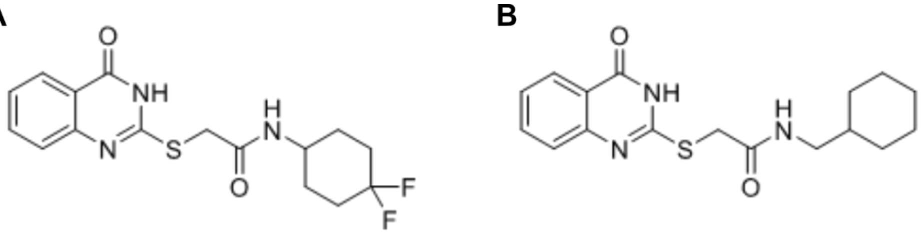

Supplementary Fig. 13. NDH-2 inhibitors. Structures of DDD00853663 **(A)** and DDD00946831 **(B)**.

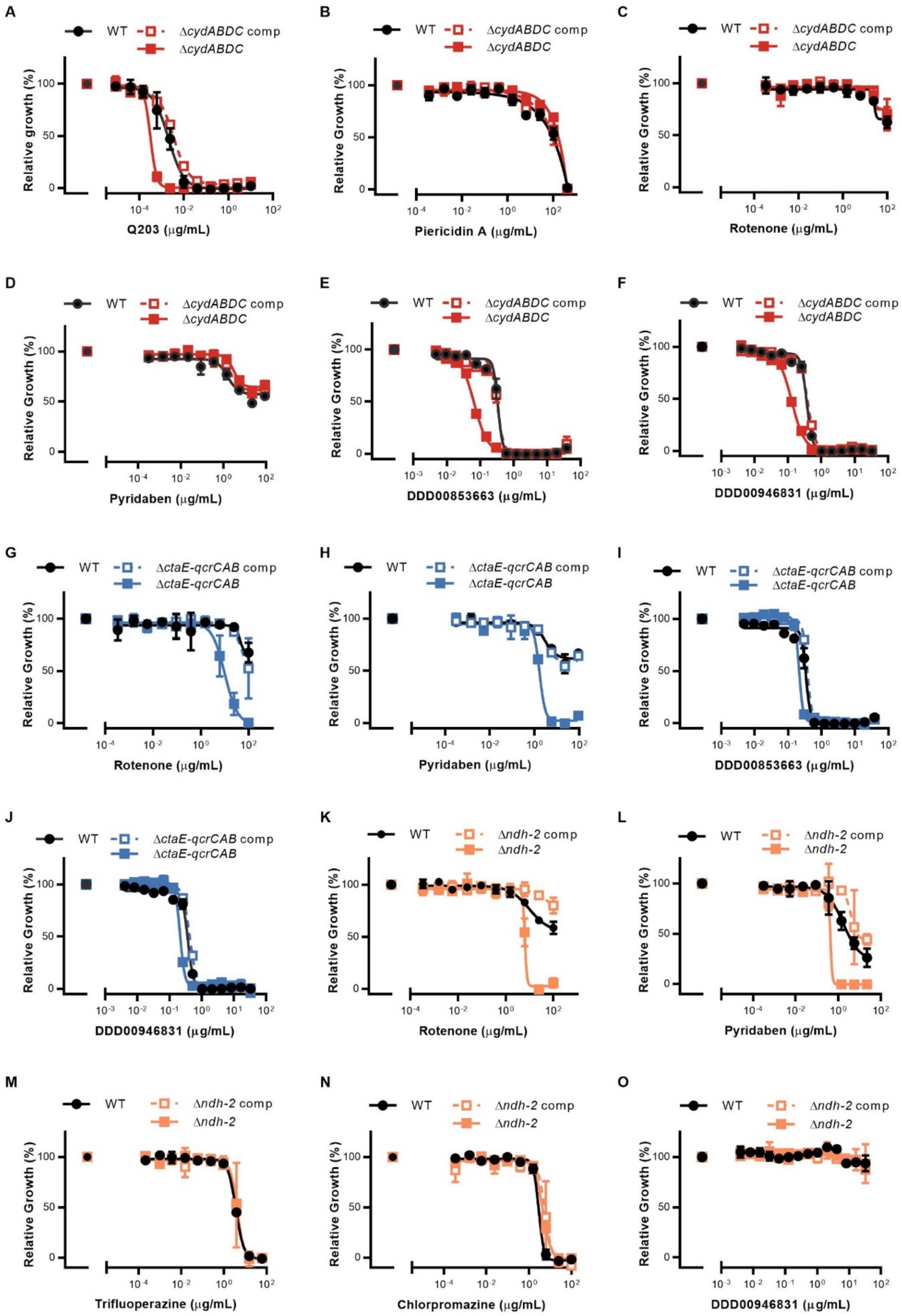

Supplementary Fig. 14. Drug susceptibilities. Impact of Q203 (**A**) piericidin A (**B**) rotenone (**C, G, K**), pyridaben (**D, H, L**), DDD00853663 (**E, I**), DDD00946831 (**F, J, O**), trifluoperazine (**M**) and chlorpromazine (**N**) on growth. Data are averages of three cultures and represent at least two independent experiments. Error bars correspond to standard deviation. “Comp” stands for complemented. Source data are provided as a Source Data file.

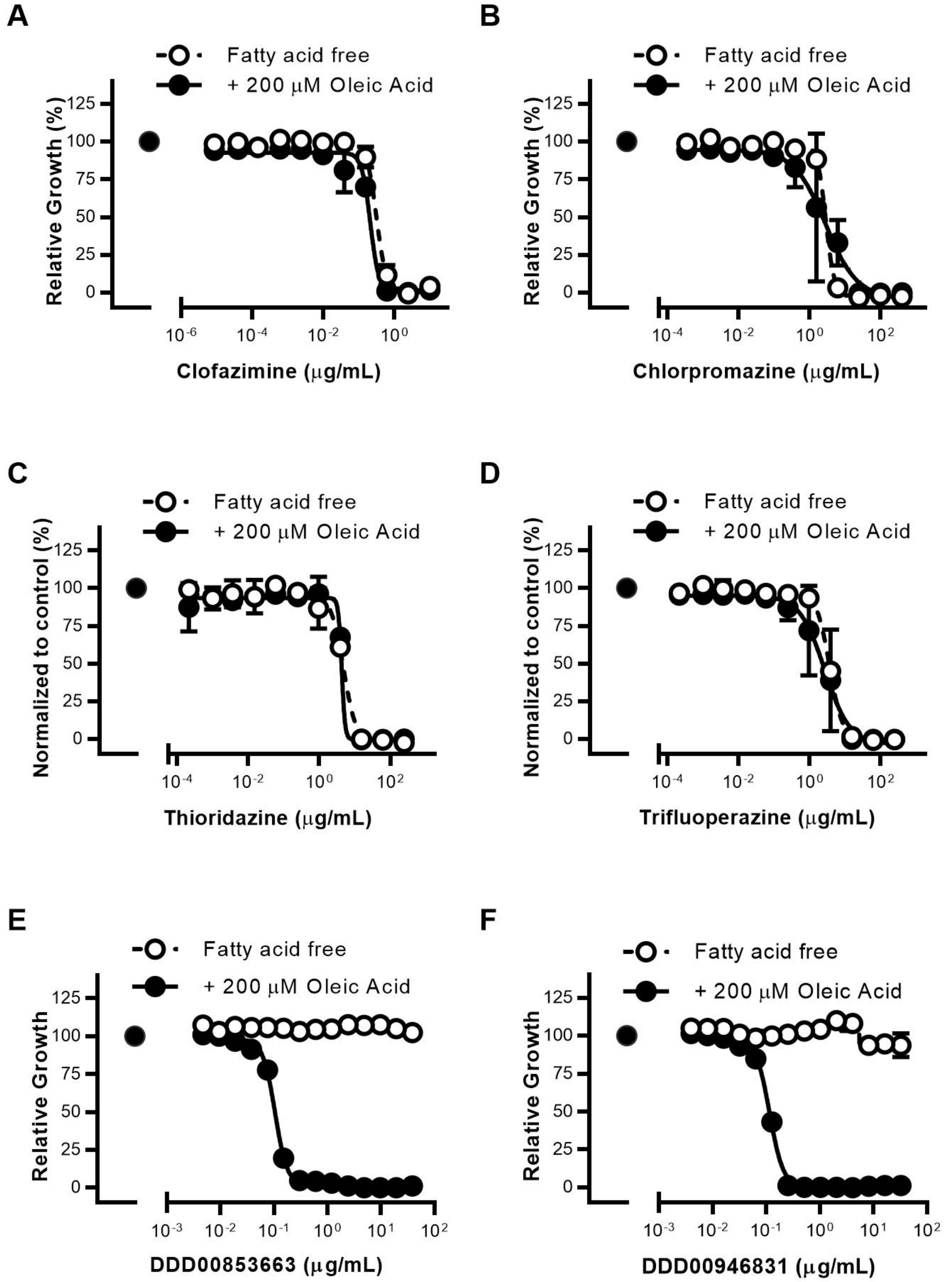

Supplementary Fig. 15. Impact of oleic acid on the activity of drugs potentially targeting RC. Profiles for clofazimine (A), chlorpromazine (B), thioridazine (C), trifluoperazine (D), DDD00853663 (E), and DDD00946831 (F). Measurements were performed in minimal media with or without oleic acid. Data are averages of three cultures and represent at least two independent experiments. Error bars correspond to standard deviation. Source data are provided as a Source Data file.

Supplementary Table 1. *Mycobacterium tuberculosis* whole genome sequencing analysis of respiratory chain knockout mutants.
Genetic polymorphisms specific to each knockout strain.

STRAIN	POSITION	GENE	DESCRIPTION	AA CHANGE	REF	ALT	# REF READS	# ALT READS	COVERAGE
Δ*ctaC*	2213712	*rv1968*	*mce3C*	I241L	A	C	0	130	130
Δ*ctaC*	2501310	*rv2226*	*rv2226*	frameshift at R372	ACG	A	0	119	119
Δ*ctaC*	3259736	*rv2933*	*ppsC*	frameshift at H895	AC	A	0	68	68
Δ*ctaD*	2856055	*rv2530A*	*vapB39*	V74A	A	G	0	109	109
Δ*ctaD*	3249676	*rv2931*	*ppsA*	frameshift at H955	A	AC	0	52	52
Δ*ctaD*	4409145	*rv3919c*	*gid*	R137stop	G	A	10	118	128
Δ*ctaE-qcrCAB*	309762	*rv0257*	Rv0257	L22V	T	G	0	170	170
Δ*ndh-2*	682776	*rv0585c*	*rv0585c*	silent	A	G	0	173	173
Δ*ndh-2*	1000403	*rv0896*	*gltA2*	R310L	G	T	0	138	138
Δ*ndh-2*	1721844	*rv1526c*	*rv1526c*	Q75stop	G	A	0	143	143

Ref- Reference sequence

Alt- Alternative sequence

Supplementary Table 2. ND10885 MIC in colonies isolated from *Mtb* infected marmoset lung lesions.

Marmoset ID	Lesion #	Colony # / Strain	MIC (μM)*
n/a	n/a	H37Rv	0.30
n/a	n/a	CDC1551	0.10
M269	8	1 - 2	0.31
		3 - 5	0.24
		6	0.31
	13	7 - 12	0.31
	19	13 -16	0.31
		17	0.39
		18	0.46
		19 -23	0.31
		24	0.3
BL24	10	25	0.31
		26	0.39
		27 -28	0.31
		29	0.30
		30	0.31
	20	31 - 36	0.31
BL131	19	37 -40	0.31
	14	41 -48	0.31
BL173	27	49 -60	0.31
	14	61 -72	0.31

* MIC was measured after one week of growth.

Supplementary Table 3. Drug susceptibility of terminal oxidase mutants in 7H9 medium.

Compound	Predicted / validated target	WT		Δ*ctaE-qcrCAB*		Δ*ctaE-qcrCAB* comp		Δ*cydABDC*		Δ*cydABDC* comp	
		MIC50	MIC	MIC50	MIC	MIC50	MIC	MIC50	MIC	MIC50	MIC
Rotenone	NDH-1	>49	>49	10-98	>25	>49	>49	>25	>49	>25	>49
Piericidin A		26-420	>100	7.1-16	23-79	100-420	>100	100-420	>100	100-420	>100
Pyridaben		>23	>23	1.5-1.9	1.9-4.3	>23	>23	>23	>23	>23	>23
Chlorpromazine	NDH-2	4.2-7.9	10-26	1.7	4.3-4.5	5.6-6.0	12-15	4.5-5.9	9.7-11	5.3-5.7	9.9-10
Thioridazine		3.4-6.5	5.2-15	2.3-3.2	4.7-6.2	4.0-4.2	5.9-11	4.3-4.7	5.7-6.5	3.7-5.7	5.9-15
Trifluoperazine		4.4-6.5	5.3-14	2.3-3.9	6.2-8.4	4.9-5.3	12-13	4.7-5.3	12-13	4.7-4.9	8.5-12
Clofazimine		0.17-0.30	0.20-0.64	0.16-0.17	0.25-0.43	0.21-0.25	0.54-0.55	0.071-0.080	0.17-0.20	0.15-0.20	0.18-0.42
DDD00853663		0.35	0.58	0.21	0.31	0.39	0.58	0.07	0.19	0.34	0.58
DDD00946831		0.36	0.60	0.22	0.30	0.43	0.70	0.13	0.36	0.39	0.66
Q203	Cytochrome bc_1 reductase	0.0019-0.016	>0.0064	>0.50	>0.50	0.00048-0.0024	>0.50	0.00031-0.00075	0.00062-0.0010	0.0011-0.0080	>0.011
Bedaquiline	ATP synthase	0.19-0.73	0.23-1.5	0.18-0.31	0.43-0.50	0.25-0.57	0.54-1.1	0.17-0.40	0.19-0.68	0.16-0.54	0.19-1.1
Valinomycin	K+ ionophore	0.44-0.58	0.62-1.4	0.39-0.63	0.66-1.5	0.66-0.99	1.5-1.9	0.42-0.47	0.95-1.2	0.54-0.57	1.4
Rifampicin	RNA polymerase	0.023-0.052	0.049-0.11	0.049-0.11	0.091-0.17	0.070-0.089	0.20-0.25	0.024-0.057	0.079-0.15	0.021-0.053	0.024-0.11
Isoniazid	InhA	0.013-0.021	0.017-0.036	0.014-0.027	0.030-0.037	0.012-0.016	0.027-0.031	0.011-0.013	0.013-0.018	0.013-0.028	0.016-0.037
Ethionamide		0.11-0.14	0.13-0.30	0.12	0.27-0.28	0.097-0.15	0.11-0.31	0.11-0.12	0.13-0.16	0.14-0.17	0.34
Ethambutol	EmbAB	0.33-0.79	0.39-1.8	0.69-1.5	1.7-1.8	0.91-1.0	2.1-2.2	0.42-0.75	0.49-1.8	0.42-0.76	0.86-1.8
Linezolid	50S ribosomal subunit	0.37-0.42	0.43-0.86	0.20-0.26	0.41-0.55	0.33-0.37	0.40-0.62	0.29-0.50	0.39-1.2	0.29-0.41	0.48-1.1
Ciprofloxacin	DNA gyrase	0.19-0.29	0.21-0.53	0.31-0.51	0.64-0.67	0.21-0.25	0.44-0.51	0.23-0.28	0.48-0.53	0.24-0.25	0.52-0.53

MIC50 Concentration (μg/mL) that inhibits bacterial growth to 50% of maximal growth and was the output IC50 value given by GraphPad Prism 7.02 when inputting the log(inhibitor) vs. response data into the software's variable slope (four parameter) nonlinear regression analysis.

MIC Lowest concentration (μg/mL) that completely inhibits bacterial growth; calculated by fitting the log(inhibitor) vs. response data to a Gompertz model using the template provided by GraphPad.

Supplementary Table 4. Drug susceptibility of Δ*ndh-2* in fatty acid free Sauton's medium.

Compound	Predicted / validated target	WT		Δ*ndh-2*		Δ*ndh-2* comp		WT (+ 200 μM Oleic acid)	
		MIC50	MIC	MIC50	MIC	MIC50	MIC	MIC50	MIC
Rotenone	NDH-1	>6.2	>25	2.9-6.3	5.9-12	>49	>49	-	-
Piericidin A		12-100	>100	0.46-1.1	0.55-1.5	100-420	>100	-	-
Pyridaben		>1.4	>23	0.43-0.77	0.52-1.2	>5.7	>23	-	-
Chlorpromazine	NDH-2	2.9-5.5	6.1-11	4.4-6.8	9.8-14	5.8-7.7	11-18	2.8-9.2	21-25
Thioridazine		4.9-5.1	12-14	4.3-6.7	5.3-15	4.6-6.9	6.3-15	4.3	5.1-8.4
Trifluoperazine		3.7-5.6	7.4-13	4.2-5.4	9.7-13	4.2-6.6	8.2-15	2.7-5.6	13-15
Clofazimine		0.32-0.71	0.67-1.3	0.16-0.51	0.25-0.94	0.55-0.69	0.67-0.83	0.21-0.50	0.56-2.2
DDD00853663		>39	>39	>39	>39	>39	>39	0.27	0.47
DDD00946831		>33	>33	>33	>33	>33	>33	0.35	0.66
Q203	Cytochrome bc_1 reductase	0.0013-0.0099	>0.0026	0.00050-0.0011	0.0010-0.0017	0.0014-0.0035	0.0029-0.0062	-	-
Bedaquiline	ATP synthase	0.22-0.50	0.33-1.0	0.044-0.15	0.051-0.17	0.24-0.38	0.49-0.58	-	-
Valinomycin	K+ ionophore	1.5-1.9	2.5-6.1	2.6-3.5	5.9-8.2	4.7-5.4	6.5-11	-	-
Rifampicin	RNA polymerase	0.031-0.050	0.078-0.11	0.093-0.16	0.27-0.38	0.14-0.20	0.28-0.42	-	-
Isoniazid	InhA	0.0072-0.0073	0.0094-0.012	0.013-0.014	0.013-0.029	0.013-0.014	0.015-0.030	-	-
Ethionamide		0.20-0.36	0.49-0.54	0.46-0.51	0.56-1.1	0.47-0.51	0.65-0.87	-	-
Ethambutol	EmbAB	0.45-0.82	1.1-1.5	0.59-1.6	1.5-3.8	1.3-1.6	2.0-2.6	-	-
Linezolid	50S ribosomal subunit	0.23-0.46	0.52-0.72	0.24-0.31	0.47-0.59	0.46	0.84-1.1	-	-
Ciprofloxacin	DNA gyrase	0.22-0.30	0.22-0.55	0.21-0.22	0.48	0.23-0.44	0.48-0.60	-	-

MIC50 Concentration (μg/mL) that inhibits bacterial growth to 50% of maximal growth and was the output IC50 value given by GraphPad Prism 7.02 when inputting the log(inhibitor) vs. response data into the software's variable slope (four parameter) nonlinear regression analysis.

MIC Lowest concentration (μg/mL, or μM*) that completely inhibits bacterial growth; calculated by fitting the log(inhibitor) vs. response data to a Gompertz model using the template provided by GraphPad.

Supplementary Table 5. Strains used in this work.

Strain	Reference
Mtb H37Rv	Trudeau Institute
Mtb Δ*ctaE-qcrCAB*::P*ctaE-qcrCAB*	This work
Mtb Δ*ctaE-qcrCAB*::*ctaE-qcrCAB*-TetOFF	This work
Mtb Δ*ctaE-qcrCAB*	This work
Mtb Δ*ctaE-qcrCAB*::complemented	This work
Mtb Δ*ctaC*::*ctaC*-DUC	This work
Mtb Δ*ctaC*	This work
Mtb Δ*ctaC*::complemented	This work
Mtb Δ*cydABDC*	This work
Mtb Δ*cydABDC* Δ*ctaE-qcrCAB*::*ctaE-qcrCAB*-TetOFF	This work
Mtb Δ*ndh/ndhA*::P*ndh*	This work
Mtb Δ*ndh/ndhA*::*ndhA*-DUC	This work
Mtb Δ*ndh/ndhA*	This work
Mtb Δ*ndh/ndhA*::complemented *ndh*	This work
Mtb Δ*ndh/ndhA*::complemented *lbnox*	This work

Supplementary Table 6. Plasmids used in this work.

Plasmid	Reference
pNit::ET	[1]
pSM270	[2]
pGMCZq17-0X0X	This work
pGMCtZq17-TSC10M1-sspB	This work
pKOts-ctaE-qcrCAB	This work
pGMCK-PctaE-qcrCAB	This work
pGMCZ-T38S38-750-ctaE-qcrCAB-SD2	This work
pGMCtKq1-PctaE-qcrCAB	This work
pGMCS-P750-*ctaC-rv2219*	This work
pGMCZ-T38S38-750-ctaC-DAS	This work
pGMCtKq1-P750-ctaC	This work
pGMCS-PctaD-serB2	This work
pGMCZ-serB2(tr)-T38S38-750-ctaD-DAS	This work
pGMCZ-PserB2	This work
pGMCtKq1-Puv15-ctaD	This work
pKO-cydABDC	This work
pGMCK-PcydABDC-SD1	This work
pMPa-Pndh	This work
pGMCS-0X750-ndhA-DAS	This work
pGMCtSq19-0X-Puv15-ndh	This work
pGMCgS-0X-Ptb38-LbNOX-FLAG-SD1	This work

Supplementary Table 7. Primers and probes used in qPCR assays

Gene	Primers/ Probes	Sequence
	atpE_Frw	GGTGATTTCGTCTGGGATGAA
atpE	atpE_Rev	GCTACCTTGCCGATCTGTTTG
	atpE probe	CGGAGGCCCTGCGTCAAGCAC
	ctaC_Frw	AACCCGGTGGCAAACAAC
ctaC	ctaC_Rev	CGCAGTGGCCCACGAATG
	ctaC probe	TCGGTCAACGTCTTCCAGATCGA
	cydA_Frw	ACTACCGGCCCAACCTCTTC
cydA	cydA_Rev	CCGGGATCGCCATCAAC
	cydA probe	CACCTACTGGTCATTTCGCATGATGATCG
	ndh_Frw	TTCGCACCCGGCATGAAG
ndh	ndh_Rev	CCGTTCGGCTTGCTCGAAAG
	ndh_probe	CCATCGACGACGCGTTGGAGTT
	ndhA_Frw	CCGCCGATGGGTCCAAAG
ndhA	ndhA_Rev	CCATCGCGTTGAGTTGAACCT
	ndhA_probe	CTGGGTCTCAAGGCACAACGGC
	nuoA_Frw	CCTGACCGCGATGTTGTTC
nuoA	nuoA_Rev	GTGCCCAGCGAGTCGTAG
	nuoA_probe	TCGACATCGAAATTGTGTTCCTCTACCC
	qcrA_Frw	GGGCCAGGAGAGTTTCAACTTC
qcrA	qcrA_Rev	TGACGGGCAACCCAAATGAG
	qcrA_probe	CGAATTCTTCGCGTTCACCAAGGTC
	sigA_Frw	GGTGATTTCGTCTGGGATGAA
sigA	sigA_Rev	GCTACCTTGCCGATCTGTTTG
	sigA_probe	CGGAGGCCCTGCGTCAAGCAC

References

1 Wei, J. R. *et al.* Depletion of antibiotic targets has widely varying effects on growth. *Proceedings of the National Academy of Sciences of the United States of America* **108**, 4176-4181, doi:10.1073/pnas.1018301108 (2011).

2 Manganelli, R., Voskuil, M. I., Schoolnik, G. K. & Smith, I. The Mycobacterium tuberculosis ECF sigma factor sigmaE: role in global gene expression and survival in macrophages. *Mol Microbiol* **41**, 423-437 (2001).
